# A Stimulation Function of Synaptotagmin-1 in Ternary SNARE Complex Formation Dependent on Munc18 and Munc13

**DOI:** 10.3389/fnmol.2017.00256

**Published:** 2017-08-15

**Authors:** Yun Li, Shen Wang, Tianzhi Li, Le Zhu, Yuanyuan Xu, Cong Ma

**Affiliations:** Key Laboratory of Molecular Biophysics of the Ministry of Education, College of Life Science and Technology, Huazhong University of Science and Technology Wuhan, China

**Keywords:** synaptotagmin-1, SNARE complex, synaptic exocytosis, membrane traffic, synaptic vesicle priming

## Abstract

The Ca^2+^ sensor synaptotagmin-1 (Syt1) plays an essential function in synaptic exocytosis. Recently, Syt1 has been implicated in synaptic vesicle priming, a maturation step prior to Ca^2+^-triggered membrane fusion that is believed to involve formation of the ternary SNARE complex and require priming proteins Munc18-1 and Munc13-1. However, the mechanisms of Syt1 in synaptic vesicle priming are still unclear. In this study, we found that Syt1 stimulates the transition from the Munc18-1/syntaxin-1 complex to the ternary SNARE complex catalyzed by Munc13-1. This stimulation can be further enhanced in a membrane-containing environment. Further, we showed that Syt1, together with Munc18-1 and Munc13-1, stimulates *trans* ternary SNARE complex formation on membranes in a manner resistant to disassembly factors NSF and α-SNAP. Disruption of a proposed Syt1/SNARE binding interface strongly abrogated the stimulation function of Syt1. Our results suggest that binding of Syt1 to an intermediate SNARE assembly with Munc18-1 and Munc13-1 is critical for the stimulation function of Syt1 in ternary SNARE complex formation, and this stimulation may underlie the priming function of Syt1 in synaptic exocytosis.

## Introduction

Neurotransmitter release by synaptic exocytosis is accomplished by the fusion of synaptic vesicles to the plasma membrane upon Ca^2+^ influx into the nerve terminal (Südhof, 2004; Rizo and Rosenmund, 2008). In contrast to most intracellular membrane fusion processes, synaptic vesicle fusion occurs in a sub-millisecond timescale in response to Ca^2+^ (Augustine et al., 1987; Südhof, 2013). To achieve this goal, most of synaptic vesicles undergo a series of maturation steps before Ca^2+^-triggered fast fusion, which include: (i) “tethering”: recruitment of synaptic vesicles to specialized sites at the presynaptic membrane called active zones (Pfeffer, 1999); (ii) “docking”: close attachment of synaptic vesicles to the fusion sites (Schimmöller et al., 1998); and (iii) “priming” that renders the docked vesicles in a semi-stable state ready for fast membrane fusion (Klenchin and Martin, 2000).

As the fusion machinery for synaptic exocytosis, SNARE proteins syntaxin-1, SNAP-25 on the presynaptic membrane and synaptobrevin-2 on synaptic vesicles are involved in vesicle docking, priming and fusion steps (Chen and Scheller, 2001; Brunger, 2005; Jahn and Scheller, 2006; Südhof and Rothman, 2009; Rizo and Xu, 2015). Syntaxin-1 initially interacts with Munc18-1, a Sec1/Munc18-like (SM) protein essential for exocytosis, to form a “closed” heterodimeric complex (Misura et al., 2000; Burkhardt et al., 2008), playing a function in vesicle docking (Voets et al., 2001; de Wit et al., 2006). Afterwards, syntaxin-1 assembles with SNAP-25 and synaptobrevin-2 into the ternary SNARE complex composed of a four-helical bundle that forces membranes into close proximity (Jahn and Scheller, 2006; Südhof and Rothman, 2009; Jahn and Fasshauer, 2012). The transition from the Munc18-1/syntaxin-1 complex to the ternary SNARE complex appears to underlie the vesicle priming reaction, where this transition is catalyzed by active zone priming factors, such as Munc13s (Augustin et al., 1999; Ma et al., 2011; Yang et al., 2015). Finally, full assembly of the ternary SNARE complex towards the C-terminal membrane anchors coincides with Ca^2+^-triggered membrane fusion and this step is highly regulated by neuronal specific proteins such as synaptotagmin-1 (Syt1) and complexins (Südhof and Rothman, 2009; Südhof, 2013; Rizo and Xu, 2015).

Syt1 is one of the major Ca^2+^ sensors that mediate synchronous neurotransmitter release (Xu et al., 2007; Bacaj et al., 2013). Syt1 anchors in the synaptic vesicle membrane via a transmembrane region, and comprises two Ca^2+^-binding C2 domains, known as C2A and C2B domains (Perin et al., 1991), which bind to acidic phospholipids in both a Ca^2+^-independent and a Ca^2+^-dependent manner (Fernandez et al., 2001; Fernández-Chacón et al., 2002). Ca^2+^-dependent binding of the C2 domains to phosphatidylserines (PS) is essential for Ca^2+^-triggered synchronous neurotransmitter release (Fernandez et al., 2001; Fernández-Chacón et al., 2002; Chapman, 2008; Bacaj et al., 2013). Moreover, recent studies positioned Syt1 as a candidate factor that regulates the maturation steps prior to Ca^2+^-triggered fusion. For instance, extensive biochemical and biophysical evidence have shown that the Ca^2+^-independent binding of the C2B domain to phosphatidylinositol 4,5-bisphosphate (PI(4,5)P2) and to the SNAREs (i.e., SNAP-25 and/or syntaxin-1) promotes docking of synaptic vesicles to the fusion sites (de Wit et al., 2009; van den Bogaart et al., 2011; Park et al., 2015).

In addition to the roles in synaptic vesicle docking and Ca^2+^-evoked fusion, Syt1 has been suggested to be critical for synaptic vesicle priming in more recent studies (Yoshihara and Littleton, 2002; Okamoto et al., 2005; Liu et al., 2009; Wang et al., 2011; Mohrmann et al., 2013; Bacaj et al., 2015). Unlike vesicle tethering and docking steps that can be morphologically assessed by measuring distances (within ~ nm ranges) between synaptic vesicles and the plasma membrane using electron microscopy (EM; Siksou et al., 2009), priming is characterized by the size of the readily releasable pool (RRP) of vesicles, which is generally measured by monitoring neurotransmitter release induced by hypertonic sucrose solution in a Ca^2+^-independent manner (Rosenmund and Stevens, 1996). In most previous observations, individual deletion of Syt1 and its isoforms (e.g., Syt2 and Syt7) exhibits no effect on the size of the RRP (Sun et al., 2007; Luo et al., 2015). However, a recent study found that simultaneous loss-of-function of both Syt1 and Syt7 dramatically decreases the RRP size in mass cultures of hippocampal neurons (Bacaj et al., 2015). Consistently, another study reported that the RRP size, the recycling pool size and release probability are all reduced when deletion of Syt1 in cultures containing multiple interconnected neurons (Liu et al., 2009). In line with these evidence, some earlier studies found that deletion of Syt1, especially its C2B domain, in *Drosophila* neuromuscular junctions (NMJs) leads to a severe reduction of the RRP size (Yoshihara and Littleton, 2002; Okamoto et al., 2005). Altogether, the RRP-promoting effect of Syt1 strongly suggests a function of Syt1 in synaptic vesicle priming. However, the underlying mechanism remains unclear.

Priming is generally believed to involve formation of the ternary SNARE complex (Sørensen et al., 2006). To explore the priming mechanism of Syt1 in synaptic exocytosis, in this study, we investigated the functional importance of Syt1 with the priming factors (i.e., Munc18-1 and Munc13-1) and the SNARE disassembly factors (i.e., NSF and α-SNAP, which are also required for priming; Xu et al., 1999; Burgalossi et al., 2010; Wickner, 2010; Ma et al., 2013) for ternary SNARE complex formation. We observed a strong Ca^2+^-independent stimulation effect of Syt1 on both *cis* and *trans* ternary SNARE complex formation in the presence of Munc18-1 and Munc13-1. In addition, this stimulation of Syt1 is resistant to the SNARE disassembly activity of NSF and α-SNAP, in a manner that critically requires Munc18-1 and Munc13-1. Our results correlate with recent *in vivo* observations that Syt1 promotes the RRP, and suggest a priming mechanism of Syt1 in synaptic exocytosis.

## Materials and Methods

### Plasmids

Rat Syt1 cytoplasmic domain (residues 140–421, known as C2AB), C2A domain (residues 140–266), C2B domain (residues 270–421) and its mutants C2B_2RQ_ (residues 270–421, R398Q and R399Q), C2b (residues 270–421, D363N and D365N), full-length rat Munc18-1, full-length synaptobrevin-2 and its cytoplasmic domain (residues 29–93), rat syntaxin-1a cytoplasmic domain (residues 2–253) and its SNARE domain (H3, residues 191–253), rat Munc13-1 MUN domain (also known as MUN^933^, residues 933–1407, EF, 1453–1531), full-length *Cricetulus griseus* NSF and full-length *Bos taurus* α-SNAP were cloned into pGEX-KG vector. Full-length human SNAP-25a (with its four native cysteines mutated to serines), its C-terminal truncation SNAP-25a Δ9 (residues 1–197, with its four native cysteines mutated to serines) and full-length rat syntaxin-1a were cloned into pET28a vector (Novagen). The co-expressed full-length rat Munc18-1 and full-length rat syntaxin-1a, full-length rat Munc18-1 and rat syntaxin-1a cytoplasmic domain (residues 1–261) were cloned to pETDuet-1 vector (Novagen). Rat Munc13-1 C_1_-C_2_B-MUN fragment (residues 529–1407, EF, 1453–1531) was cloned into pFastBac™HtB vector (Invitrogen). All of the mutants used in this study were generated by using QuikChange Site-Directed Mutagenesis Kit (Stratagene).

### Recombinant Protein Expression and Purification

Rat Munc13-1 C_1_-C_2_B-MUN fragment was expressed in Sf9 insect cells as previous described (Ma et al., 2013). All other proteins were expressed in *E. coli* BL21 DE3 by culturing the cells to 0.6–0.8 O.D._600_ at 37°C, then induced with 0.4 mM isopropyl-β-D-thiogalactoside (IPTG, Amresco) for 16–20 h at 20°C. Cultured cells were harvested by centrifuging at 4200 rpm in a J6-MI centrifuge equipped with JS-4.2 rotor (Beckman Coulter) at 4°C.

For GST-tagged proteins, cell pellets from 1 L of culture were resuspended with 50 mM Na_2_HPO_4_-NaH_2_PO_4_ pH 7.6, 300 mM NaCl, 10% (v/v) glycerol, 0.5% Triton X-100 (Sigma; lysis buffer A) supplied with 1 mM phenylmethanesulfonyl fluoride (PMSF, Amresco) and 5 mM 2-mercaptoethanol (2-ME, Amresco). Cells were broken using an AH-1500 Nano Homogenize Machine (ATS Engineering Inc.) at 1200 bar for three times at 4°C. Cell lysates were centrifuged at 16,000 rpm in a JA-25.50 rotor (Beckman Coulter) at 4°C. The supernatants were collected and mixed with 1 ml glutathione Sepharose 4B (GE Healthcare) affinity media. After 2 h rotation at 4°C, the mixture were washed twice with lysis buffer A supplied with 2 mM 2-ME. For purification of GST-syntaxin-1a H3 domain, GST-Munc18-1 and GST-MUN, proteins were eluted by a buffer containing 50 mM Tris-Cl, 300 mM NaCl, 10% (v/v) glycerol and 20 mM L-glutathione (GSH, Amresco), pH 8.0. Eluted proteins were desalted by PD-10 desalting column (GE Healthcare) using a buffer containing 25 mM HEPES-KOH, 150 mM KCl and 10% (v/v) glycerol, pH 7.6 (buffer H) supplied with 0.2 mM tris(2-carboxyethyl)phosphine (TCEP, Sigma). For purification tag-free proteins, GST-tag were removed by mixing 10 U/ml of thrombin (from bovine pancreatic) into the media in buffer H supplied with 0.2 mM TCEP at 4°C overnight. Eluted proteins were further purified by ion exchange chromatography (Source 15S/15Q, GE Healthcare) followed by size-exclusion chromatography (Superdex 75 pg/200 pg, GE Healthcare). For hexa-histidine tagged proteins, cell pellets from 1 L of culture were resuspended with 50 mM Tris-Cl pH 8.0, 300 mM NaCl, 10% (v/v) glycerol, 0.5% Triton X-100 (Sigma; lysis buffer B) supplied with 1 mM PMSF and 2 mM 2-ME. Cells were broken using an AH-1500 Nano Homogenize Machine (ATS Engineering Inc.) at 1200 bar for three times at 4°C. Cell lysates were centrifuged at 16,000 rpm in a JA-25.50 rotor (Beckman Coulter) at 4°C. The supernatants were collected and mixed with 1 ml Nickel-NTA agarose (Qiagen) affinity media. After 2 h rotation at 4°C, the mixture were washed twice with lysis buffer B supplied with 1 mM 2-ME and 30 mM imidazole followed by an additional wash step with Triton X-100-free lysis buffer B supplied with 1 mM 2-ME and 30 mM imidazole. Proteins were eluted with a buffer containing 20 mM Tris-Cl pH 8.0, 150 mM NaCl, 10% (v/v) glycerol (buffer T) supplied with 300 mM imidazole and 0.2 mM TCEP. For full-length syntaxin-1a and co-expressed full-length Munc18-1/syntaxin-1, proteins were eluted with buffer T supplied with 300 mM imidazole, 0.2 mM TCEP and 1% (w/v) sodium cholate (Aladdin, Shanghai, China). Eluted proteins were desalted by using PD-10 desalting column (GE Healthcare) and further purified by ion exchange chromatography (Source 15Q, GE Healthcare) followed by size-exclusion chromatography (Superdex 75 pg/200 pg, GE Healthcare).

Purification buffers for proteins with transmembrane domain were supplied with 1% (w/v) n-octyl-β-D-glucoside (β-OG, Amresco; for full-length synaptobrevin-2) or 1% (w/v) sodium cholate (Aladdin, Shanghai, China; for full-length syntaxin-1a and co-expressed full-length Munc18-1/syntaxin-1). Protein purities were checked using SDS-PAGE (>95%) and final concentrations were determined by A_280_ on a P330 NanoPhotometer (Implen).

### Fluorescent Labeling of Purified Proteins

BODIPY FL [N-(2-aminoethyl)maleimide] (BDPY, Molecular Probes) and tetramethylrhodamine-5-maleimide, single isomer (TMR, Molecular Probes) were used as fluorescence resonance energy transfer (FRET) donor and acceptor in this study. Dye-protein conjugation was achieved by maleimide-cysteine conjugation (all cysteine mutants for dye conjugation were described in the figure). Powdered dyes were dissolved by using dimethylsulfoxide (DMSO, Sigma; for BDPY) or dimethyl formamide (DMF, Aladdin, Shanghai, China; for TMR) to a concentration of 40 mM and stored at −20°C. Purified proteins were dialyzed with buffer H containing 0.2 mM TCEP and 0.2 mM EDTA and diluted to a concentration of 20–40 μM. Dyes were gently added to the protein solutions with a protein-to-dye ratio of 1:5. Dye-protein mixtures were gently rotated overnight at 4°C in the dark room. Excess dyes were bleached by adding 10 mM dithiothreitol (DTT, Amresco) and removed by using PD-10 desalting column (GE Healthcare). Labeling/conjugation efficiency was calculated by:

(1) $$\text{E}=\frac{A_{\text{D}}*\varepsilon_{\text{p}}}{\varepsilon_{\text{D}}*(A_{280}-A_{\text{D}}*C_{280})}*100\%$$

where *A*_D_ is the peak absorbance of the dye; *A*_280_ is the absorbance of the dye-protein compound at 280 nm; *ε*_p_ is the extinction coefficient of the protein at 280 nm; *ε*_D_ is the extinction coefficient of the dye at the absorption maximum; *C*_280_ is the so called dye correction factor since some dyes display an absorbance at 280 nm. The *C*_280_ is given as:

(2) $$C_{\text{280}}=\varepsilon_{\text{D280}}/\varepsilon_{\text{D}}$$

where *ε*_D280_ is the extinction coefficient of the dye at 280 nm. About 90% of the labeling efficiency was reached for each samples.

### GST Pull-Down Assay

Purified GST-syntaxin-1a H3 domain (residues 191–253) was incubated with purified SNAP-25a and the cytoplasmic domain of synaptobrevin-2 (residues 29–93) at 4°C overnight. The sample was analyzed by SDS-PAGE to confirm the SNARE complex formation. For GST pull-down assay, 20 μl 50% (v/v) glutathione Sepharose 4B affinity media (GE Healthcare) was mixed with 5 μM GST-SNARE complex, 5 μM GST-Munc18-1, 5 μM GST-Munc13-1 MUN domain (residues 933–1407, EF, 1453–1531), 5 μM GST, respectively and 10 μM Syt1 C2B domain to a final volume of 50 μl. The samples were gently rotated for 2 h at 4°C followed by washing with buffer H three times. Finally, the samples were analyzed by SDS-PAGE followed by coomassie brilliant blue staining and immunoblotting (anti-Syt1, rabbit polyclonal, Proteintech, Wuhan, China). All of the experiments were performed in the absence of Ca^2+^.

### Liposome Preparation

1-palmitoyl-2-oleoyl-*sn*-glycero-3-phosphocholine (POPC), 1-palmitoyl-2-oleoyl-*sn*-glycero-3-phosphoethanolamine (POPE), 1,2-dioleoyl-*sn-*glycero-3-phospho-L-serine (sodium salt; DOPS), L-α-phosphatidylinositol-4,5-bisphosphate (Brain, Porcine; ammonium salt; PI[4,5]P2), cholesterol (ovine wool; Chol) and 1-2-dioleoyl-*sn*-glycerol (DAG) were all obtained from Avanti Polar Lipids. T-liposome reconstituted with Munc18-1/syntaxin-1 and t’-liposome containing syntaxin-1/SNAP-25 Δ9 comprises 37% POPC, 20% POPE, 20% DOPS, 2% DAG, 1% PI(4,5)P2 and 20% Chol and v-liposome reconstituted with BDPY-labeled synaptobrevin-2 comprises 45% POPC, 20% POPE, 15% DOPS and 20% Chol, which are demonstrated in the schematic diagrams in each figures. Lipid mixtures in glass tubes were dried under nitrogen gas flow in water bath at 40°C followed by vacuuming in a vacuum desiccator for 3 h. Lipid films were dissolved in buffer H containing 0.2 mM TCEP and 1% (w/v) sodium cholate. After vortexing for 5 min, purified proteins (i.e., full-length syntaxin-1a, full-length Munc18-1/syntaxin-1 or BDPY-labeled full-length synaptobrevin-2) were added into the mixture to a protein-to-lipid ratio of 1:200. An additional round of vortex were performed to mix the proteins and lipid mixture. The proteoliposomes were formed by desalting the protein-lipid mixtures with PD-10 desalting column (GE Healthcare) in buffer H containing 0.2 mM TCEP. To remove any residual detergents and contaminants, a routine dialysis step was performed with buffer H containing 0.2 mM TCEP, 0.2 mM EDTA and 0.5 g/L Bio-beads SM2 (Bio-Rad).

### Fluorescence Experiments

All of the FRET assays in this study were carried out on a PTI QM-40 spectrophotometer with excitation and emission wavelength of 485 nm and 513 nm, respectively at 37°C.

**Figure 1:**
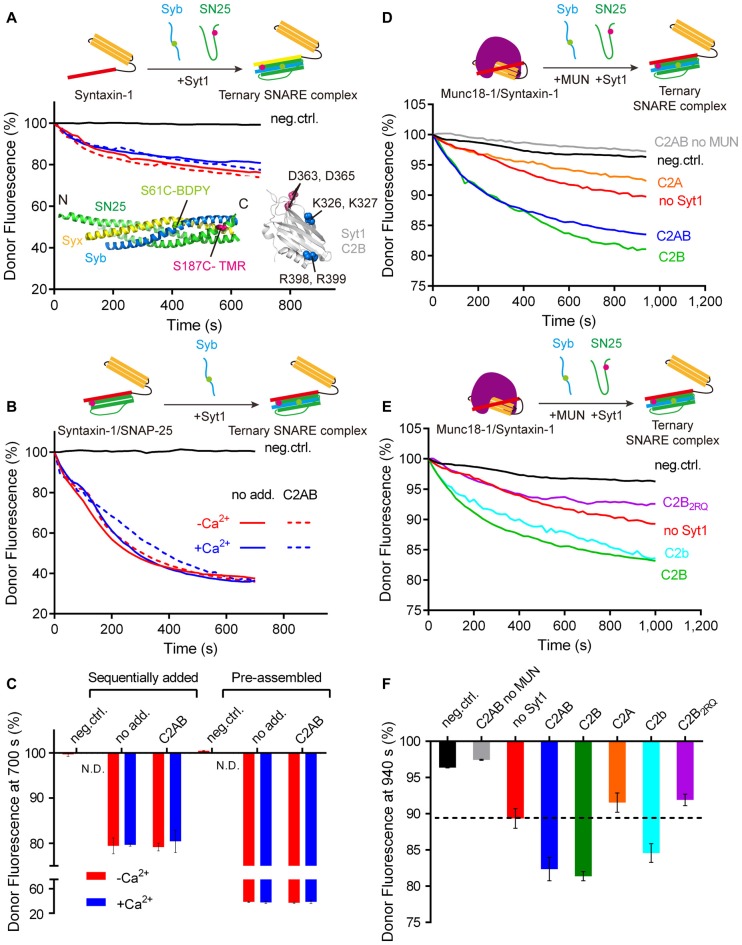
Synaptotagmin-1 (Syt1) stimulates ternary SNARE complex formation with Munc18-1 and Munc13-1. **(A,B)** Syt1 actions on ternary SNARE complex formation started with isolated syntaxin-1 and SNAP-25 **(A)** and preassembled syntaxin-1/SNAP-25 heterodimers **(B)** in the absence (0.2 mM EDTA) and presence of 1 mM Ca^2+^. Inset displays a fluorescent-labeling scheme of the SNARE complex (PDB entry 1N7S) and Syt1 C2B domain (PDB entry 1TJX) with indicated poly-basic stretch (K326, K327), bottom face (R398, R399) and Ca^2+^-binding sites (D363, D365).** (C)** Quantification of the results in **(A,B)**. **(D)** Syt1 stimulates ternary SNARE complex formation with Munc18-1 and the MUN domain through its C2B domain in the absence of Ca^2+^ (0.2 mM EDTA). **(E)** The bottom face (i.e., R398, R399) of Syt1 is essential for stimulating ternary SNARE complex formation with Munc18-1 and the MUN domain in the absence of Ca^2+^ (0.2 mM EDTA). **(F)** Quantification of the results in **(D,E)**. Schematic diagrams were displayed on the top of each charts. Representative traces from one of three independent experiments are shown. Data in each bar chart were presented as means ± SD, *n* = 3, technical replicates. neg.ctrl., excess unlabeled cytoplasmic domain of synaptobrevin-2 was incorporated. Syb, synaptobrevin-2; SN25, SNAP-25.

For Syt1 stimulating ternary SNARE complex formation with and without Munc18-1 and Munc13-1 in membrane-free system (Figure 1 and Supplementary Figure S1), 5μM syntaxin-1a cytoplasmic domain (residues 2–253) were mixed with 5 μM TMR-labeled SNAP-25a (S187C), 1 μM BDPY-labeled synaptobrevin-2 cytoplasmic domain (residues 29–93, S61C) and 2 μM Syt1 fragment. Alternatively, pre-assembled syntaxin-1a cytoplasmic domain (residues 2–253) and TMR-labeled SNAP-25a (S187C) were added to 5 μM as well. Five micromolar co-expressed transmembrane-free Munc18-1/syntaxin-1 (residues 1–261) were mixed with 5 μM TMR-labeled SNAP-25a (S187C), 1 μM BDPY-labeled synaptobrevin-2 cytoplasmic domain (residues 29–93, S61C), 30 μM Munc13-1 MUN domain (residues 933–1407, EF, 1453–1531) and 2 μM Syt1 fragment. CaCl_2_ were added, if indicated, at a final concentration of 1 mM.

**Figure 2:**
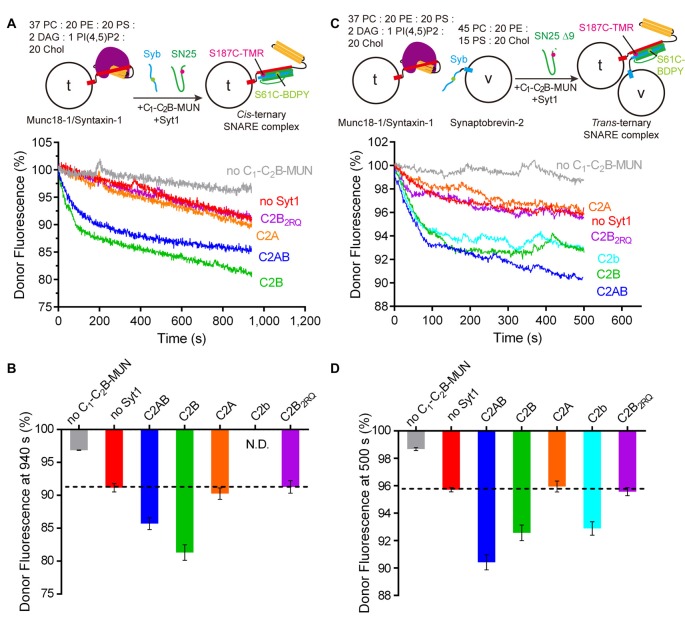
Syt1 stimulates *cis-* and *trans-* ternary SNARE complex formation with Munc18 and Munc13-1 in the presence of the membranes.** (A)** Syt1 promotes *cis* ternary SNARE complex formation between membrane-embedded Munc18-1/syntaxin-1 complex (t-liposomes) and cytoplasmic domain of synaptobrevin-2 in the presence of SNAP-25, the Munc13-1 C_1_-C_2_B-MUN fragment and 0.2 mM EDTA.** (B)** Quantification of the results in **(A)**.** (C)** Syt1 stimulates *trans* ternary SNARE complex formation between membrane-embedded Munc18-1/syntaxin-1 complex (t-liposomes) and membrane-embedded synaptobrevin-2 (v-liposomes) in the presence of SNAP-25 Δ9, the Munc13-1 C_1_-C_2_B-MUN fragment, 0.2 mM EDTA.** (D)** Quantification of the results in **(C)**. Schematic diagrams and lipid compositions were displayed on the top of each charts. Representative traces from one of three independent experiments are shown. Data in the bar charts were presented as means ± SD, *n* = 3, technical replicates.

**Figure 3:**
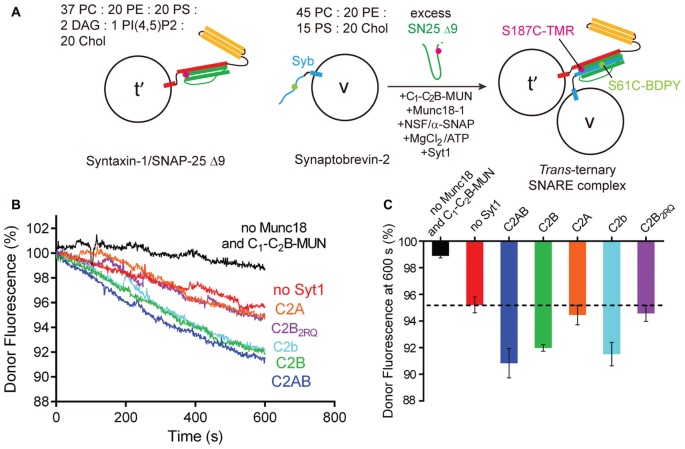
Syt1 stimulates *trans* ternary SNARE complex formation in the presence of Munc18-1, Munc13-1, NSF and α-SNAP.** (A)** Schematic diagram of *trans* ternary SNARE complex formation between syntaxin-1/SNAP-25 Δ9 liposomes (t’-liposomes) and synaptobrevin-2 liposomes (v-liposomes) in the presence of Munc18-1, the Munc13-1 C_1_-C_2_B-MUN fragment, NSF, α-SNAP, ATP, Mg^2+^ and 0.2 mM EDTA.** (B)** Syt1 promotes *trans-*ternary SNARE complex formation in the presence of Munc18-1, Munc13-1 C_1_-C_2_B-MUN fragment, NSF, α-SNAP, ATP, Mg^2+^ and 0.2 mM EDTA.** (C)** Quantification of the results in **(B)**. Lipid compositions were displayed on the top of the schematic diagram. Representative traces from one of three independent experiments are shown. Data in the bar chart was presented as means ± SD, *n* = 3, technical replicates.

For Syt1 stimulating ternary SNARE complex formation with Munc18-1 and Munc13-1 in the presence of membranes (Figures 2, 3), general procedures are the same to those in membrane-free system. Particularly, Munc13-1 MUN domain was replaced by Munc13-1 C_1_-C_2_B-MUN fragment (0.5 μM) and the amount of Syt1 fragments were reduced to 0.5 μM due to the recruiting effect of PI(4,5)P2 on t-liposomes. For *trans* SNARE complex formation, TMR labeled SNAP-25a Δ9 (residues 1–197, S187C) was applied. 0.4 μM full-length NSF, 1 μM α-SNAP, 2 mM ATP-2Na^+^ (Sangon, Shanghai, China) and 2 mM MgCl_2_ were incorporated as indicated. 0.2 mM EDTA was present all the time to chelate residual Ca^2+^ in the reaction buffer.

### Graphing and Mathematical Methods

Prism 6.01 (GraphPad) was used to graph and perform non-linear curve fits.

## Results

### Syt1 Stimulates Ternary SNARE Complex Formation with Munc18-1 and Munc13-1

As introduced above, synaptic vesicle priming is generally believed to involve ternary SNARE complex formation. However, the action of Syt1 in ternary SNARE complex formation has been unclear. To this aim, we first examined whether the cytoplasmic fragment of Syt1 (referred to as C2AB) stimulates formation of the ternary SNARE complex in solution. Fluorescence donor and acceptor were separately labeled to synaptobrevin-2 (residues 29–93, S61C) and SNAP-25 (S187C), respectively, and ternary SNARE complex formation was measured by a decrease of donor fluorescence based on FRET (Figure 1A). Addition of C2AB did not influence formation of the ternary SNARE complex in the absence and presence of Ca^2+^ (Figures 1A,C). Similarly, C2AB did not affect formation of synaptobrevin-2 with preassembled syntaxin-1/SNAP-25 heterodimers in the absence and presence of Ca^2+^ (Figures 1B,C). These results are consistent with previous observations (Stein et al., 2007; Chicka et al., 2008), suggesting that Syt1 has no obvious effect on ternary SNARE complex formation when beginning merely with the three SNAREs.

In a physiological context, SNARE complex formation is highly regulated by Munc18-1 and Munc13-1, the two key factors required for synaptic vesicle priming (Augustin et al., 1999; Südhof and Rothman, 2009). We therefore explored whether Syt1 stimulates ternary SNARE complex formation in the presence of Munc18-1 and Munc13-1 using the FRET assay. As observed in our previous studies (Ma et al., 2011, 2013; Yang et al., 2015), the MUN domain, which contains the minimal priming activity of Munc13-1 (Basu et al., 2005), catalyzed the transition from the Munc18-1/syntaxin-1 complex to the ternary SNARE complex (Figures 1D,F). Intriguingly, further addition of C2AB strongly enhanced this transition in a Ca^2+^-independent manner (Figures 1D,F and Supplementary Figure S1), whereas this enhancement was not observed when the MUN domain was absent (Figures 1D,F). These results indicate that Syt1 stimulates ternary SNARE complex formation in a Munc18/Munc13-dependent manner.

We further identified the minimal requirement of Syt1 for its stimulation activity. We found that the C2B domain was sufficient for the stimulation, whereas the C2A domain was not (Figures 1D,F). These results suggest that the C2B domain mediates the stimulation activity of Syt1 in ternary SNARE complex formation dependent on Munc18-1 and Munc13-1.

Structurally, the C2B domain comprises several conserved basic residues at the “bottom” (the R398 R399 region) and at the “side” (a poly-basic stretch, referred to as the K326 K327 region; Figure 1A). Abundant positive charges contributed by these two basic regions endow the C2B domain with the ability to bind acidic SNARE complexes and acidic phospholipids, which helps to couple the functions of Syt1 and the SNAREs in synaptic exocytosis (Mohrmann et al., 2013; Brewer et al., 2015; Zhou et al., 2015; Wang et al., 2016). The K326 K327 region was implicated in recruitment of Syt1 onto the PI(4,5)P2-enriched plasma membrane (van den Bogaart et al., 2011; Park et al., 2015), whereas the R398 R399 region has been recently found to interact with the SNAREs and the SNARE complex (Zhou et al., 2015; Wang et al., 2016).

Therefore, we asked whether disruption of the R398 R399 region that is supposed to impair the SNARE binding affects the stimulation activity of the C2B domain. Indeed, mutation of the R398 R399 region of the C2B domain (R398Q/R399Q, referred to as C2B_2RQ_) impeded the stimulation activity of the C2B domain in MUN-catalyzed transition from the Munc18-1/syntaxin-1 complex to the ternary SNARE complex (Figures 1E,F). In contrast, mutation of the Ca^2+^-binding sites of the C2B domain (D363N/D365N, referred to as C2b) had no such effect (Figures 1E,F). These results suggest that the C2B/SNARE interaction is important for the stimulation function of Syt1 in ternary SNARE complex formation dependent on Munc18-1 and Munc13-1.

On the other hand, we also tested the interactions of the C2B domain with the MUN domain and/or Munc18-1 for its stimulation activity. However, no detectable binding between the C2B domain and the MUN domain or Munc18-1 can be observed using GST pull-down assay in solution (Supplementary Figure S2). Despite that, considering that the stimulation effect of the C2B domain on ternary SNARE complex formation strictly requires both Munc18-1 and Munc13-1 (Figure 1D), there might have weak and cooperative interactions among the C2B domain, Munc18-1 and Munc13-1, which orchestrate the C2B/SNARE interaction. Therefore, we expected that, in the presence of membranes, the interactions among the above proteins would be increased due to the protein-recruitment function of several lipid molecules (e.g., PI(4,5)P2 and PS; Martin, 2015; Pinheiro et al., 2016), resulting in enhanced stimulation activity of Syt1 in SNARE complex formation.

### The Stimulation Effect of Syt1 Is Enhanced in the Presence of Membranes

We next explored the stimulation activity of Syt1 in the transition from the Munc18-1/syntaxin-1 complex to the ternary SNARE complex in a membrane-containing environment. To this goal, we developed a membrane-based FRET assay in which the Munc18-1/syntaxin-1 complex was reconstituted into liposomes (referred to as t-liposomes), whereas synaptobrevin-2 was present either in solution (using the cytoplasmic domain of synaptobrevin-2 (residues 29–93, S61C), see Figure 2A) or reconstituted onto liposomes (using full-length synaptobrevin-2 (residues 1–116, S61C), referred to v-liposomes, see Figure 2C). Note that, *cis* ternary SNARE complex formation was monitored by using SNAP-25 (S187C) and the cytoplasmic domain of synaptobrevin-2 in the presence of t-liposomes (Figure 2A); in contrast, full-length synaptobrevin-2 and a C-terminal truncated SNAP-25 fragment (residues 1–197, S187C, referred to as SNAP-25 Δ9) were applied to monitor* trans* ternary SNARE complex formation between t- and v-liposomes (Figure 2C), because SNAP-25 Δ9 can assemble into the *trans* ternary SNARE complex without inducing membrane fusion (Binz et al., 1994; Lu, 2015). Fluorescence donor and acceptor were separately labeled to synaptobrevin-2 and SNAP-25 as shown in Figures 2A,C, respectively. In this assay, a Munc13-1 C_1_-C_2_B-MUN fragment (which includes the C_1_ and C_2_B domains that bind to DAG and PI(4,5)P2 on t-liposomes, respectively), rather than the MUN domain, was applied, due to its high ability for membrane binding (Ma et al., 2013; Liu et al., 2016). Consistent with the results observed in solution (Figures 1D–F), we observed that both C2AB and C2B of Syt1 were able to stimulate the transition of the Munc18-1/syntaxin-1 complex to the ternary SNARE complex on membranes with the addition of the C_1_-C_2_B-MUN fragment and SNAP-25 (or SNAP-25 Δ9), no matter synaptobrevin-2 was present in a soluble state (Figures 2A,B) or embedded in the membrane (Figures 2C,D). As control, this stimulation was abrogated in the absence of the C_1_-C_2_B-MUN fragment (Figures 2A–D). In addition, C2B_2RQ_ lost the stimulation activity (Figures 2A–D). These data suggest that Syt1 stimulates both *cis* and *trans* ternary SNARE complex formation on membranes in the presence of Munc18-1 and Munc13-1.

We next analyzed the kinetics of the Syt1-stimulated transition reactions in the absence and presence of membranes (Table 1 and Supplementary Figure S3). Compared to membrane-free system (Supplementary Figure S3A), one-membrane system (i.e., t-liposomes and the cytoplasmic domain of synaptobrevin-2, see Supplementary Figure S3B) exhibited comparable reaction rate in the absence of C2AB (1.299 ± 0.100 × 10^−3^ s^−1^ vs. 1.352 ± 0.039 × 10^−3^ s^−1^), but displayed faster rate in the presence of C2AB (3.540 ± 0.090 × 10^−3^ s^−1^ vs. 15.20 ± 0.69 × 10^−3^ s^−1^; Table 1). This acceleration arises likely from robust association of C2AB with membranes via binding to PI(4,5)P2, thus increasing C2AB binding probability to the SNAREs, Munc18-1 and Munc13-1 on the membrane. In contrast to the membrane-free and one-membrane systems, two-membrane system (i.e., t-liposomes and v-liposomes, see Supplementary Figure S3C) showed even higher reaction rate both in the absence and presence of C2AB (12.29 ± 1.76 × 10^−3^ s^−1^ and 24.93 ± 1.02 × 10^−3^ s^−1^, respectively; Table 1), which arises likely because the C_1_-C_2_B-MUN fragment efficiently associates t- and v-liposomes into close proximity and thus further increases collision probabilities among these proteins (Liu et al., 2016). These results suggest that the membrane behaves as a perfect platform, enabling Syt1 cooperation with Munc18-1 and Munc13-1 to stimulate *trans* ternary SNARE complex formation more efficiently.

**Table 1:** Data summary of the non-linear curve fits of synaptotagmin-1-stimulated SNARE complex formation in membrane-free, one-membrane and two-membrane systems.

	$k_{\text{fast}}^{†}$ * 10^−3^ (s^−1^)		$\tau_{\text{fast}}^{‡}$ (s)		R-square	
	No Syt1	Syt1 C2AB	No Syt1	Syt1 C2AB	No Syt1	Syt1 C2AB
Membrane-free	1.299 ± 0.100	3.540 ± 0.090	769.7	282.5	0.9948	0.9970
t- only	1.352 ± 0.039	15.20 ± 0.69	739.8	65.78	0.9749	0.9868
t- and v-	12.29 ± 1.76	24.93 ± 1.02	81.36	40.12	0.9739	0.9863

*^†^Rate constant for fast phase; ^‡^Time constant for fast phase. Data plots were fitted to two-phase exponential decay function except for “membrane-free”, which was fitted to one-phase exponential decay function. The data were presented as means ± SE*.

### Syt1 Stimulates *Trans* SNARE Complex Formation in the Presence of Munc18-1, Munc13-1, NSF and α-SNAP

It was previously shown that, started with liposomes containing syntaxin-1/SNAP-25 heterodimers, the efficient lipid mixing with synaptobrevin-2 liposomes in the presence of Ca^2+^ and Syt1 (C2AB) was abolished by incorporation of NSF and α-SNAP because they disassemble syntaxin-1/SNAP-25 heterodimers (Ma et al., 2013; Liu et al., 2016). In the presence of NSF and α-SNAP, efficient membrane fusion requires both Munc18-1 and Munc13-1 (the C_1_-C_2_B-MUN fragment) to orchestrate SNARE-mediated membrane fusion in an NSF/α-SNAP-resistant manner (Ma et al., 2013; Liu et al., 2016). These results suggest that assembly and disassembly factors (i.e., Munc18-1/Munc13-1 and NSF/α-SNAP, respectively) function in alternation to catalyze cycles of membrane fusion (Hughson, 2013; Ma et al., 2013; Rizo and Xu, 2015). In this regard, we investigated whether Syt1 stimulates *trans* ternary SNARE complex formation starting with syntaxin-1/SNAP-25 heterodimers in the presence of Munc18-1, Munc13-1, NSF and α-SNAP in a membrane-containing environment.

To monitor the formation of *trans* ternary SNARE complexes on membranes, we routinely used synaptobrevin-2 (full-length, S61C) and SNAP-25 Δ9 (S187C) as FRET donor and acceptor, respectively (Figure 3A). Mixing syntaxin-1/SNAP-25 Δ9 liposomes (t’-liposomes) and synaptobrevin-2 liposomes (v-liposomes) produced *trans* ternary SNARE complex formation in the presence of Munc18-1, Munc13-1 (the C_1_-C_2_B-MUN fragment), NSF, α-SNAP, ATP and Mg^2+^ (Figures 3B,C). This result is in agreement with the notion that Munc18-1 captures the syntaxin-1 from the membrane-embedded syntaxin-1/SNAP-25 heterodimer assisted by NSF and α-SNAP, leading to the transition of the Munc18-1/syntaxin-1 complex to the ternary SNARE complex catalyzed by Munc13-1 through an NSF/α-SNAP-resistant pathway (Ma et al., 2013). Intriguingly, we observed that both C2AB and C2B, instead of C2A, were able to further stimulate *trans* ternary SNARE complex formation in the presence of Munc18-1, Munc13-1, NSF and α-SNAP (Figures 3B,C). In contrast, C2B_2RQ_, but not C2b, abolished this stimulation (Figures 3B,C), reinforcing the importance of the C2B/SNARE interaction for the stimulation function of Syt1. However, the stimulation of Syt1 was abrogated when Munc18-1 and Munc13-1 were absent (Figures 3B,C), as NSF and α-SNAP disassemble the syntaxin-1/SNAP-25 complex to prevent SNARE complex formation (Supplementary Figure S4). These results imply that Syt1 cooperates with Munc18-1 and Munc13-1 to stimulate* trans* ternary SNARE complex on membranes in an NSF/α-SNAP-resistant manner.

### Syt1 Might Stabilize a Ternary SNARE Assembly with Munc18-1 and Munc13-1

**Figure 4:**
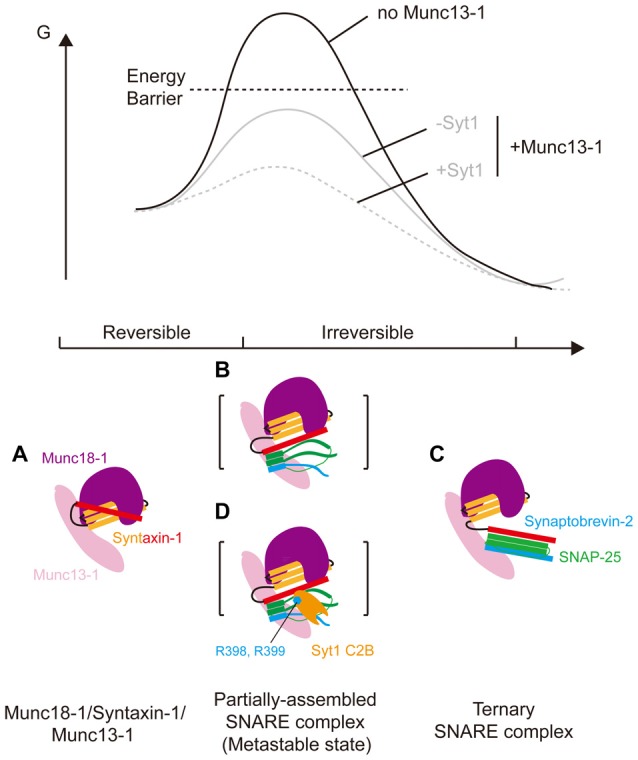
Energy landscape of the transition from the Munc18-1/syntaxin-1 complex to the ternary SNARE complex in the presence of Munc13-1 and Syt1. **(A)** Binding of Munc13-1 to the Munc18-1/syntaxin-1 complex induces a conformational rearrangement in the syntaxin-1 linker region (low Gibbs free energy) thus **(B)** providing a potential nucleation site for N-terminal assembly among syntaxin-1, SNAP-25 and synaptobrevin-2 (metastable state with rather high Gibbs free energy, which is below the energy barrier (black dashed line)); **(C)** propagation of the four helical SNARE bundle toward the C-terminal end would eventually dissociate the H3 domain of syntaxin-1 from Munc18-1 to reach a stable ternary SNARE complex (extremely low Gibbs free energy). **(D)** Binding of Syt1 C2B domain to the partially-assembled ternary SNARE complex (metastable state) would help to lower the Gibbs free energy of the total system (gray solid line to gray dashed line), thus stimulating the reaction. Munc18-1, SNAP-25, synaptobrevin-2, Munc13-1, Syt1 C2B domain are colored in purple, green, blue, pink and orange, respectively. Syntaxin-1 is colored in yellow/black/red (yellow: the Habc domain; black: the linker region; red: the H3 domain). The bottom face of Syt1 C2B domain (i.e., R398, R399) is displayed as blue hexagon.

We recently found that binding of Munc13-1 to the Munc18-1/syntaxin-1 complex induces a conformational rearrangement in the syntaxin-1 linker region that cause higher reactivity towards ternary SNARE complex formation (Wang et al., 2017; Figure 4A). With Munc13-1, transit of syntaxin-1 from the Munc18-1/syntaxin-1 complex to the ternary SNARE complex strictly requires the simultaneous existence of SNAP-25 and synaptobrevin-2 (Wang et al., 2017). It is thus conceivable that, the conformational change in the syntaxin-1 linker region induced by Munc13-1 would expose the N-terminal end of the H3 domain of syntaxin-1, providing a nucleation site for SNAP-25 and synaptobrevin-2 binding (Figure 4B). Subsequently, propagation of the four helical SNARE bundle toward the C-terminal end would eventually dissociate the H3 domain from the closed Munc18-1/syntaxin-1 complex (Figure 4C). This model challenges the current dogma on the role of the steadily preassembled syntaxin-1/SNAP-25 heterodimer (“t-SNARE”) as an “on-pathway” product in neuronal SNARE complex formation (Bhalla et al., 2006; Wiederhold and Fasshauer, 2009; Hui et al., 2011), suggesting a simultaneous “N-to-C” assembly of the three SNAREs chaperoned by Munc18-1 and Munc13-1 (Figures 4A–C). Based on this model and our present results, we hypothesize that, binding of Syt1 to a partially installed ternary SNARE assembly with Munc18-1 and Munc13-1 (Figure 4D), promotes SNARE zippering forward to the C-terminal end of the ternary SNARE complex. Indeed, this hypothesis is supported by our observation that disruption of the C2B/SNARE interaction by introducing the R398Q/R399Q mutations abolishes the stimulation function of Syt1 in ternary SNARE complex formation dependent on Munc18-1 and Munc13-1 (Figures 1–3).

## Discussion

Recent *in vivo* studies suggested a function of Syt1 in synaptic vesicle priming (Yoshihara and Littleton, 2002; Okamoto et al., 2005; Liu et al., 2009; Wang et al., 2011; Mohrmann et al., 2013; Bacaj et al., 2015), but the underlying mechanism of Syt1 is still a mystery. In this study, we observed a strong stimulation activity of Syt1 in ternary SNARE complex formation in the presence of Munc18-1 and Munc13-1. We suggest that this stimulation function of Syt1 may underlie the action of Syt1 in synaptic vesicle priming.

Previous *in vitro* reconstitution studies found that Syt1 strongly promotes SNARE-dependent membrane fusion in response to Ca^2+^ (Tucker et al., 2004; Bhalla et al., 2006; Xue et al., 2008; van den Bogaart et al., 2011; Wang et al., 2011). One aspect of this promotion was suggested to involve an ability of Syt1 in accelerating assembly of syntaxin-1/SNAP-25 heterodimers or the SNARE complex on membranes in a Ca^2+^-dependent manner (Bhalla et al., 2006; Lai et al., 2014). However, this result is not consistent with *in vivo* observations that Syt1 promotes the RRP in a Ca^2+^-independent manner (Bacaj et al., 2015). In addition, although syntaxin-1/SNAP-25 heterodimers appears to be highly reactive when pairing with synaptobrevin-2 for membrane fusion (in the presence of Syt1 and Ca^2+^), recent *in vitro* studies indicated that fusion is totally abolished when the disassembly factors NSF and α-SNAP are included (Ma et al., 2013; Liu et al., 2016). Therefore, the actual neuronal SNARE complex formation pathway need to be reconsidered, with increasing evidence indicative of the vital functions of Munc18-1 and Munc13-1 in protecting ternary SNARE complex formation (Ma et al., 2013; Liu et al., 2016).

Our finding that Syt1 efficiently stimulates Munc13-catalyzed transition from the Munc18-1/syntaxin-1 complex to the ternary SNARE complex in the absence of Ca^2+^ (Figures 1D–F) correlates well with a recently proposed SNARE complex formation pathway (Ma et al., 2013). In this pathway, the Munc18-1/syntaxin-1 complex, rather than the syntaxin-1/SNAP-25 heterodimer, is suggested to represent the physiologically relevant starting point for ternary SNARE complex formation and membrane fusion. In our present study, we showed that the Ca^2+^-independent stimulation effect of Syt1 in *trans* ternary SNARE complex formation on membranes is totally blocked in the presence NSF and α-SNAP, but can be restored with the addition of Munc18-1 and Munc13-1 (Figures 3B,C). These data reinforce the chaperon function of Munc18-1 and Munc13-1 in protecting SNARE-mediated membrane fusion (Hughson, 2013; Ma et al., 2013), and suggest that in this Munc18-1/Munc13-1-chaperoned SNARE complex assembly pathway, the vital role of Syt1 in promoting ternary SNARE complex assembly can be effectively reconstituted.

In our proposed model (Figure 4), we suggest that Syt1 acts cooperatively with Munc18-1 and Munc13-1 for its function in promoting ternary SNARE complex formation with the following reasons: (i) the stimulation function of Syt1 in ternary SNARE complex formation can only be observed in the presence of Munc18-1 and Munc13-1 (Figure 1); (ii) this stimulation can be further enhanced in the presence of the membranes (Figure 2), arising likely because Munc13-promoted association of the membranes, together with PI(4,5)P2-mediated recruitment of multiple priming factors, induces high efficiency of protein-protein assembly; and (iii) multiple transient and low affinity interactions can provide high avidity and specificity, while maintaining the reversibility necessary to orchestrate dynamic assemblies (Figure 4).

A recent crystallographic study revealed three distinct Syt1/SNARE binding interfaces; among which the largest one (“the primary interface” that involves the conserved residues R398 and R399 at the bottom of the C2B domain) interacts with several negatively charged residues on SNAP-25 and syntaxin-1 (residues D51, E52, E55 on SNAP-25 and D231, E234, E238 on syntaxin-1, respectively) and plays a key function in Ca^2+^-triggered release (Xue et al., 2008; Zhou et al., 2015). Our present work extends this evidence, suggesting an important role of R398 and R399 in synaptic vesicle priming, as mutation of R398 and R399 abolishes the stimulation function of Syt1 in ternary SNARE complex formation dependent on Munc18-1 and Munc13-1 (with and without NSF and α-SNAP; Figures 1–3). Moreover, we suspect that Munc18-1 and Munc13-1 likely help to render the C2B domain of Syt1 in a proper configuration, leaving the bottom face of the C2B domain (R398 R399) in close contact with the SNARE four-helical bundle (Figure 4D, see also the region II of the priming interface in the Syt1/SNARE structure; Zhou et al., 2015).

In addition to the functional interplay of Syt1 with Munc18-1 and Munc13-1 in ternary SNARE complex formation we described above, Syt1 was found to stabilize and promote ternary SNARE complex formation with complexins (Bacaj et al., 2015) reported in a recent study using a reduced system (i.e., in HEK293T). Moreover, it was suggested that Syt1 induces multimeric assemblies of the ternary SNARE complex in a Ca^2+^-independent manner (Zhou et al., 2015). All of these activities of Syt1 may contribute to promoting synaptic vesicle priming.

In conclusion, our present study suggests a molecular mechanism of Syt1 in SNARE complex formation that couples its actions in synaptic vesicle priming. Future *in vivo* research will be required to investigate whether R398 and R399 are involved in promoting the RRP size that underlies synaptic vesicle priming, and decipher the membrane-reconstituted Munc13-1/Munc18-1/Syt1/SNARE assembly that underlies the priming state.

## Author Contributions

YL, SW and CM designed the study and analyzed the data; wrote the article. YL, SW, TL, LZ and YX purified the proteins. YL and SW performed the experiments. CM supervised the study.

## Conflict of Interest Statement

The authors declare that the research was conducted in the absence of any commercial or financial relationships that could be construed as a potential conflict of interest.
