# Supplementary material for: A Stimulation Function of Synaptotagmin-1 in Ternary SNARE Complex Formation Dependent on Munc18 and Munc13
Source: Front Mol Neurosci. 2017 Aug 15;10:256. doi: 10.3389/fnmol.2017.00256 (PMC5559510; doi:10.3389/fnmol.2017.00256)
Supplement: Supplementary file 1 [file Data_Sheet_1.doc]

### Supplementary Figures


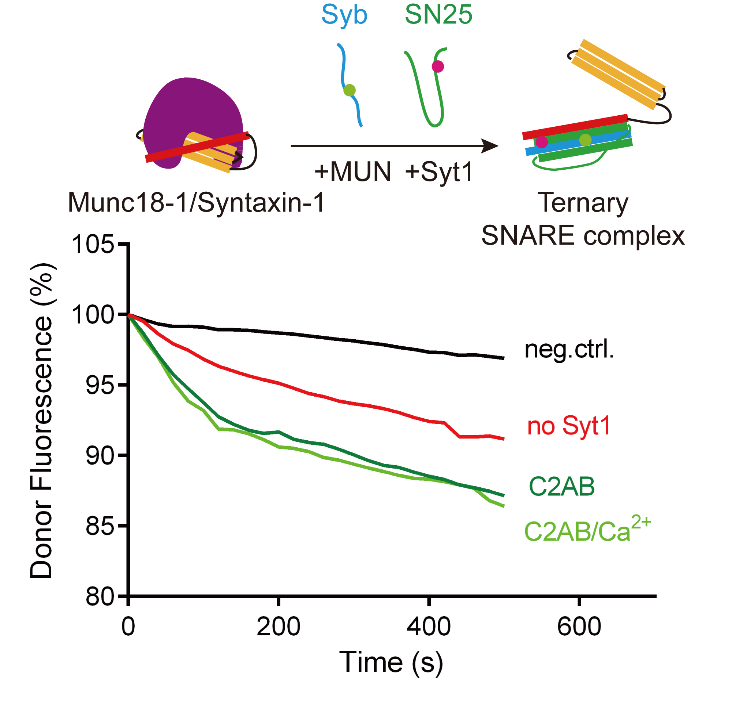


**Supplementary** **Figure 1. The stimulation function of Syt1 does not rely on Ca^2+^.** Schematic diagram was displayed on the top of the chart. Representative traces from one of three independent experiments are shown. neg.ctrl., excess unlabeled cytoplasmic domain of synaptobrevin-2 was incorporated.


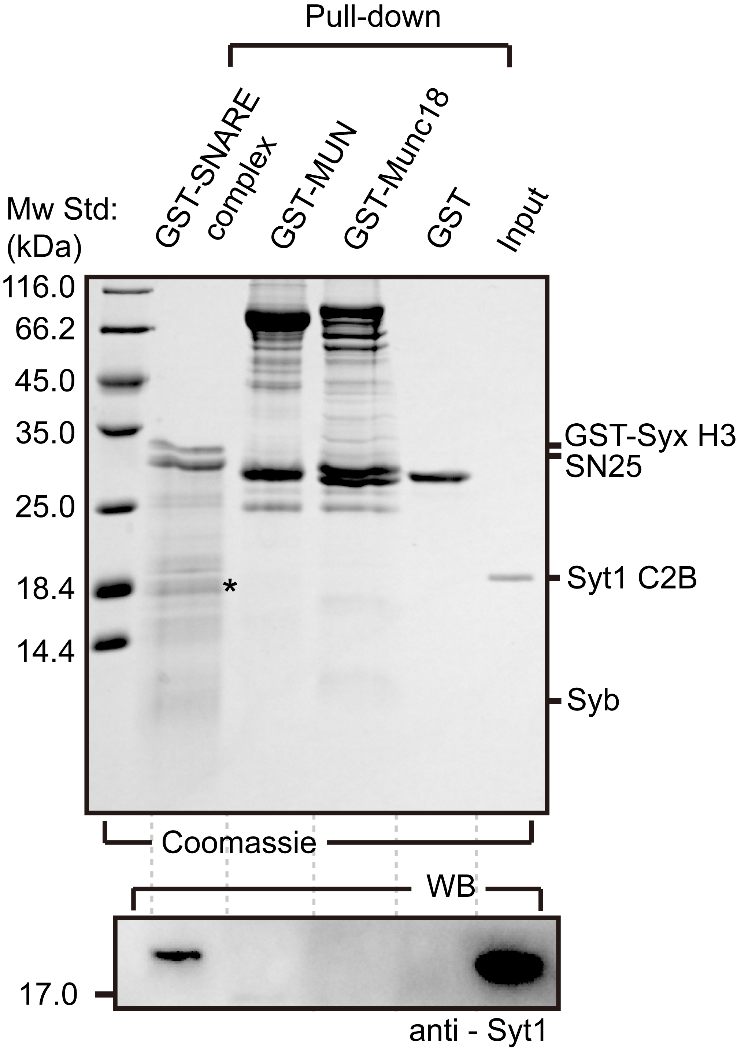


**Supplementary** **Figure 2. No detectable binding can be observed between Syt1 and Munc18-1 or the MUN domain.** Binding of the Syt1 C2B domain with assembled core SNARE complex (containing the H3 domain but not the H_abc_ domain of syntaxin-1), Munc18 and Munc13-1 MUN domain were measured by GST pull-down assay. The samples were analyzed by SDS-PAGE followed by coomassie brilliant blue staining and immunoblotting. Asterisk shows the band of C2B.


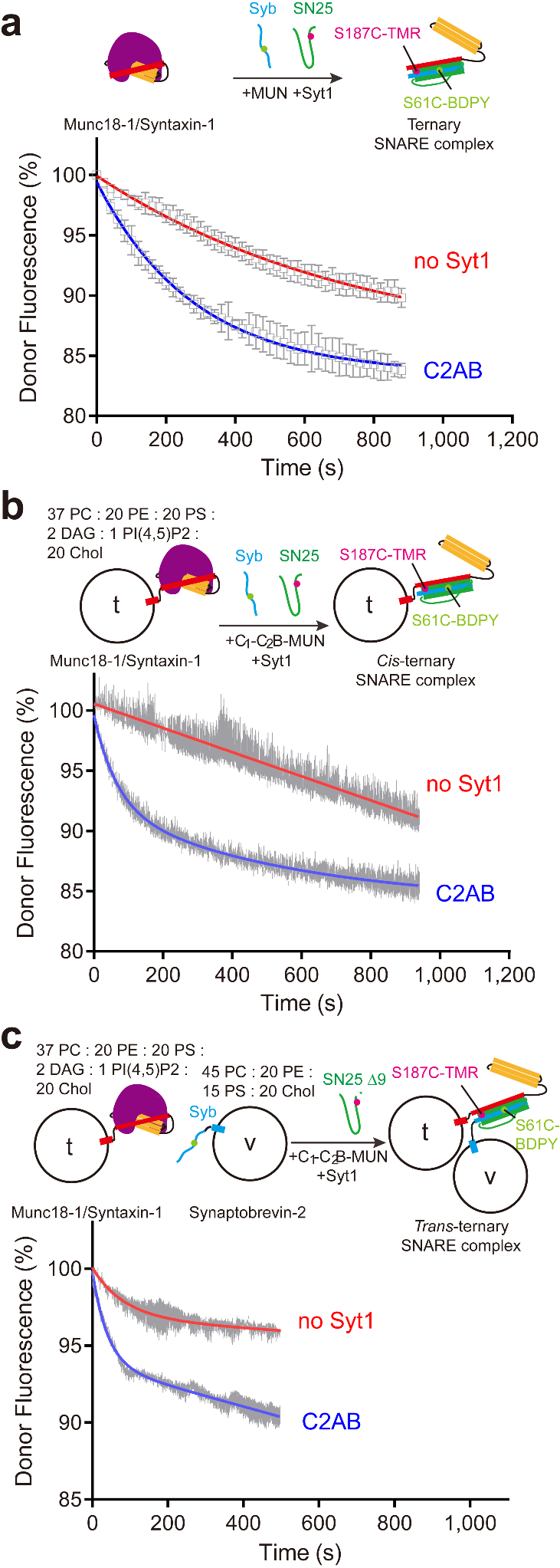


**Supplementary** **Figure 3. Syt1 stimulation efficiency in membrane-free, one-membrane and two-membrane system.** (**a–c**) Efficiency of Syt1 C2AB stimulation in membrane-free (**a**), one-membrane (t-liposomes and cytoplasmic domain of synaptobrevin-2) (**b**) and two-membrane (t-liposomes and v-liposomes) system (**c**). The traces were fitted to one-phase exponential decay (**a**) and two-phase exponential decay (**b, c**) functions, respectively. Schematic diagrams was displayed on the top of the charts. Data plots were displayed as means ± SD, n = 3, technical replicates.


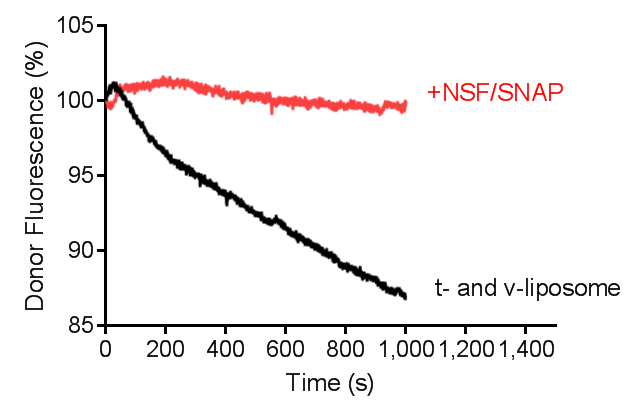


**Supplementary** **Figure 4. NSF and α-SNAP prevent membrane-embedded SNARE complex assembly.** Representative traces from one of three independent experiments are shown.
